# CONSORT-Characteristics and metabolic phenotype of gut microbiota in NAFLD patients

**DOI:** 10.1097/MD.0000000000029347

**Published:** 2022-06-24

**Authors:** Haize Ge, Wei Wei, Liang Tang, Yaqiong Tian, Yu Zhu, Yan Luo, Shuye Liu

**Affiliations:** a Department of Clinical Laboratory, the Third Central Hospital of Tianjin, Tianjin Key Laboratory of Extracorporeal Life Support for Critical Diseases, Artificial Cell Engineering Technology Research Center, Tianjin Institute of Hepatobiliary Disease, Tianjin, China; b Department of Severe Hepatitis, Tianjin Second People's Hospital, Tianjin Medical Institute of Hepatology, Tianjin, China; c Department of Osteology, Tianjin Haihe Hospital, Tianjin, China; d Department of Clinical Laboratory, Tianjin Haihe Hospital, Tianjin, China; e Department of Gastroenterology, Tianjin Haihe Hospital, Tianjin, China.

**Keywords:** fatty liver, gut microbiota, metabolism, xanthine oxidase

## Abstract

Patients with nonalcoholic fatty liver disease (NAFLD) have symptoms of a gut microbiota disorder with abnormal amino acid and glycolipid metabolism. This study was designed to analyze the characteristics of gut microbiota in patients with NAFLD, predict the gut microbiota phenotype, explore its role in the diagnosis of NAFLD, and establish its significance in disease progression.

The characteristics of the gut microbiota in NAFLD patients (n = 28, 45.8 ± 14.2 years, male/female = 18/10) and healthy subjects (n = 20, 49.6 ± 4.8 years, male/female = 14/6) during March–May 2020 were analyzed using 16S rRNA sequencing technology and the phenotypes with large differences were predicted using the Tax4Fun method. The metabolites in the fecal samples of the patients were analyzed using mass spectrometry, and their correlation with different microorganisms was examined. The accuracy of the gut microbiota in diagnosing NAFLD was investigated by receiver operating characteristic curve analysis.

We found that the microbial diversity and Bacteroides/Firmicutes (BF) ratio changed significantly (*P* < .05) in the feces of NAFLD patients. Phenotypic prediction showed that there were significant differences in the phenotypes of amino acid, glucose, and lipid metabolism of gut microbiota in the NAFLD group (*P* < .05). receiver operating characteristic curve analysis revealed that combination of Bacteroides and the BF ratio resulted in 88% and 100% sensitivity and specificity, respectively, when used for NAFLD diagnosis. Metabolomics and bioinformatics analysis revealed changes in the metabolism of nicotinate, nicotinamide, and pyrimidine; signaling pathways of calcium and oxytocin; pancreatic secretion with metabolites such as uracil, xanthine, and biliverdin; and enzymes such as xanthine dehydrogenase and xanthine oxidase (*P* < .05).

Therefore, the phenotypic changes may be a potential marker for NAFLD and we considered that a combined analysis of Bacteroides and BF ratio had good diagnostic accuracy for NAFLD.

## 1 Introduction

Fatty liver is a clinicopathological syndrome characterized by hepatocyte steatosis and lipid accumulation caused by a variety of conditions with advanced pathological stages, including simple fatty liver, steatohepatitis, fatty liver fibrosis, and fatty cirrhosis.^[1]^ Nonalcoholic fatty liver disease (NAFLD), characterized by excessive fat deposition in liver cells caused without alcohol or other direct liver-harming factors, has emerged as an important cause of chronic liver disease in developed countries such as Europe and the USA. With improvements in living standards, lifestyle changes, and increased obese populations, NAFLD is becoming increasingly common in China.^[2]^

Many studies have confirmed that the gut microbiota affects the disease occurrence and development.^[3]^ The gut of healthy people contains more than 1,000 different bacteria with approximately ten times more number of cells than those making up the entire human body.^[4]^ The gut microbiota plays an important role in body fat storage, energy metabolism, inflammatory responses, and immune regulation. Their mechanism of action is possibly associated with endotoxemia, endogenous ethanol levels, disturbances in bile metabolism, intestinal short-chain fatty acid levels, and gastrointestinal hormone secretion.^[5]^

Additionally, changes in the gut microbiota and its products are associated with the pathogenesis of NAFLD. It acts on different receptors and pathways affecting the metabolism of trimethylamine, secondary bile acids, acetic acid, propionic acid, and endogenous ethanol, which can promote NAFLD pathogenesis.^[6]^ However, in-depth studies are required to establish the role of gut microbiota and its effects on metabolism in NAFLD.

We proposed a hypothesis that there is a correlation between pathogenic bacterium/probiotics and different metabolites resulting from abnormal gut microbiota in patients with NAFLD. Additionally, we attempted to identify a diagnostic marker for NAFLD.

The present study analyzed the microbial diversity and Bacteroides/Firmicutes (BF) ratio, in which lower diversity and higher BF ratio were correlated with NAFLD symptoms and abnormal metabolism. We predicted the phenotype of gut microbiota in NAFLD patients and its correlation with the level of metabolites in their feces. Consequently, we explored the mechanism by which NAFLD affects metabolism and observed that abnormal pancreatic secretion, pyrimidine, purine metabolism, and oxidation-reduction balance are important phenotypic changes in NAFLD.^[7]^ We also investigated the possibility of using the gut microbiota as a diagnostic target for NAFLD by receiver operating characteristic curve (ROC) analysis of bacteria.

## 2 Methods

### 2.1 General information

A total of 28 Chinese patients diagnosed with NAFLD (NAFLD group) at the Third Central Hospital of Tianjin during March–May 2020 and 20 healthy volunteers (normal group) participated in the study. Their fecal samples were collected and stored at -80°C for 16S rRNA sequencing, liquid/gas chromatography-mass spectrometry (LC/GC-MS), or ELISA analysis. This study was approved by the Medical Ethics Committee of the Third Central Hospital of Tianjin according to the Declaration of Helsinki, and the patients provided informed consent before the experiments (IRB2021-038-01). There were no significant differences between the NAFLD and normal groups with respect to baseline data of sex and age; however, liver damage indices such as γ-glutamine transferase (GGT), alanine aminotransferase (ALT), and aspartate aminotransferase were increased in the NAFLD group, as shown in Table 1.

**Table 1:** Summary of clinical parameters of all individuals.

Phenotype	Normal	NAFLD	*P*-value
Sex (male/female)	14/6	18/10	1.0000
Age (year)	49.6 ± 4.8	45.8 ± 14.2	.2541
GGT (U/L)	25.8 ± 10.5	57.1 ± 23.3	<.0001
ALT (U/L)	18.8 ± 5.3	45.2 ± 23.0	<.0001
AST (U/L)	21.3 ± 3.8	33.5 ± 17.1	.003

AST = aspartate aminotransferase, GGT = γ-glutamine transferas.

#### 2.1.1 Inclusion criteria

1. No history of alcohol consumption or <140 g and <70 g alcohol equivalent per week for men and women, respectively; 2. No history of viral hepatitis, drug-induced liver disease, total parenteral nutrition, hepatolenticular degeneration, or any other specific diseases that can lead to fatty liver; 3. No signs of fatigue, dyspepsia, liver pain, hepatosplenomegaly, and other non-NAFLD specific symptoms; 4. Overweight and/or visceral obesity, increased fasting blood glucose, dyslipidemia, hypertension, and other metabolic syndrome-related components; 5. Slightly to moderately increased (less than five times the upper limit of the normal value) levels of serum transaminase and GGT, and most prominently, increased ALT levels; 6. Liver imaging findings in accordance with the imaging diagnostic criteria for diffuse fatty liver: 7. Histological changes in the liver biopsy in accordance with the pathological diagnostic criteria for fatty liver disease.

#### 2.1.2 Exclusion criteria

Long-term drinking history, generally for more than 5 years, equivalent to 80 g per day over the course of 2 weeks or 40 g and 20 g per day for men and women, respectively; 2. Viral hepatitis; 3. A history of autoimmune liver disease and/or hereditary disease; 4. A history of drug-induced liver disease; and 5. History of total parenteral nutrition, 6. A history of diabetes or hyperthyroidism; 7. Current use of any drug affecting insulin secretion or insulin sensitivity; 8. Malignant tumors and other progressive fatal diseases.

### 2.2 16S rRNA sequencing analysis

Total RNA was extracted from 300 mg of feces, and the variable region V4 of the 16S gene was PCR-amplified using a fecal genome extraction kit. The primers used were: 515F (5- GTGCCAGCMGCCGCGGTAA-3′) and 805R (5’-GGACTACHVGGGTWTCTAAT-3’). The PCR reaction was set up as follows: 94°C initial denaturation for 2 minutes; 94°C for 30 seconds, 72°C for 45 seconds for total 30 cycles; 72°C final extension for 5 minutes. Using the TruSeqDNA PCR-Free Sample Preparation Kit library kit (Illumina Company) for library construction, V4 bacterial rRNA genes were sequenced using the HiSeq2500PE250 platform.^[8]^

### 2.3 Analysis of metabolic and enzyme composition

Fresh feces sample (100 mg) was collected and placed in 80% (v/v) cold methanol and the sample was homogenized, followed by centrifugation at 13,000 × g for 15 min at 4°C. The supernatant was collected and frozen. Later, 50 μL of methylamine pyridine solution (20 mg/mL) were added and the mixture was placed in a water bath at 4°C for 2 hour with 40 μL MSTFA salinization. It was centrifuged at 13,000 × g for 15 minutes at 4°C. The sample was subjected to LC/GC-MS and nuclear magnetic resonance analysis (Thermo Fisher). The raw data files generated by UHPLC-MS/MS were processed using Compound Discoverer 3.0 (CD3.0, Thermo Fisher). The levels of xanthine dehydrogenase and xanthine oxidase in feces supernatant were measured by ELISA using Multiskan GO microplate reader (Thermo Fisher).

### 2.4 Clinical phenotype analysis

Venous blood samples were collected from individuals on an empty stomach and centrifuged at 300 × g for 10 minutes at room temperature. Serum ALT, aspartate aminotransferase, and GGT levels were estimated using a Roche Cobas8000 automatic biochemical analyzer.

### 2.5 Statistical methods

Sequencing, metabolic, and ELISA data were collected using the HiSeq2500PE250 platform, LC/GC-MS, and microplate reader respectively. Data were expressed as x ± s, using SPSS software (version 11.0; SPSS, Inc., Chicago, IL). One-way analysis of variance was used to compare groups and multiple comparisons between groups were performed using LSD *t*-tests. Turkey and Wilcoxon rank-sum tests were used to analyze differences in species beta diversity. Spearman's correlation analysis was performed using GraphPad Prism software version 8.0. The structural heterogeneity of the gut microbiota was analyzed by principal coordinates analysis (PCoA), and the gut microbiota network analysis was performed using R software (Version 2.15.3). The metabolite-enzyme network was constructed using Cytoscape 3.8.0 software. Differences were considered statistically significant at *P* < .05. All experiments were replicated thrice for the accuracy and reproducibility of results.

## 3 Results

### 3.1 Changes in gut microbiota diversity in NAFLD patients

**Figure 1:**
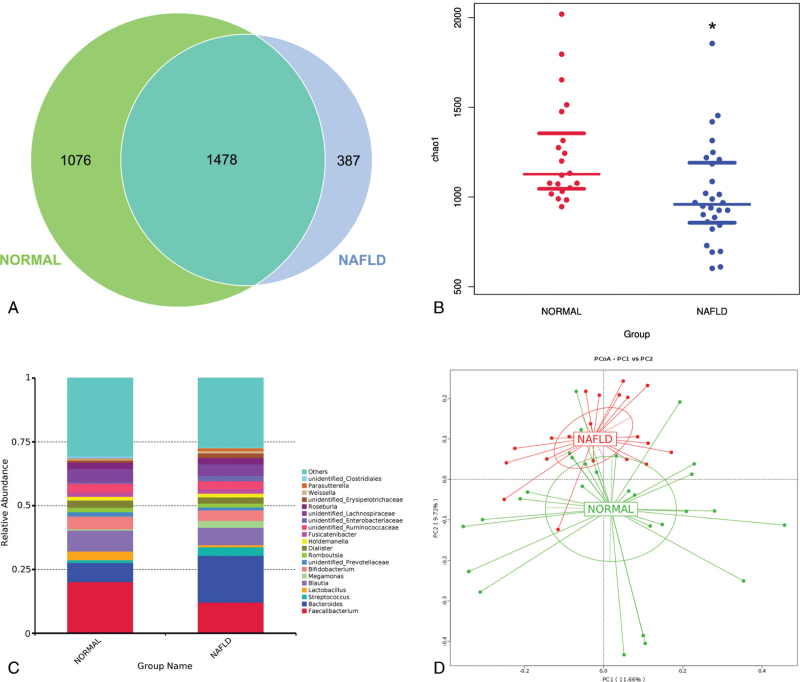
The characteristics of gut microbiota in NAFLD patients. (A) The Venn analysis showed that there were 1,076 unique and 387 unique OTUs in Normal and NAFLD group. (B) The Chao1 analysis showed a decreased alpha diversity in NAFLD group than that in Normal group. (C) The relative abundance of gut microbiota of Normal and NAFLD group in genus. (D) The PCoA analysis of gut microbiota of the Normal and NAFLD group.

The Venn graph results showed that the normal and NAFLD groups had 1,076 and 387 unique OTUs, respectively (Fig. 1A). Chao1 analysis showed that the alpha diversity of the NAFLD group decreased, indicating that the richness and diversity of the microbial communities in the NAFLD group were lower than those in the normal group (Fig. 1B). The relative abundance changes in the top 20 genera are shown in Fig. 1C. PCoA analysis showed that there was a difference in beta diversity between the normal and NAFLD groups. While the normal group had a more concentrated microbial community structure, the NAFLD group showed a scattered pattern (Fig. 1D).

### 3.2 Prediction of gut microbiota phenotypes in the NAFLD patients

**Figure 2:**
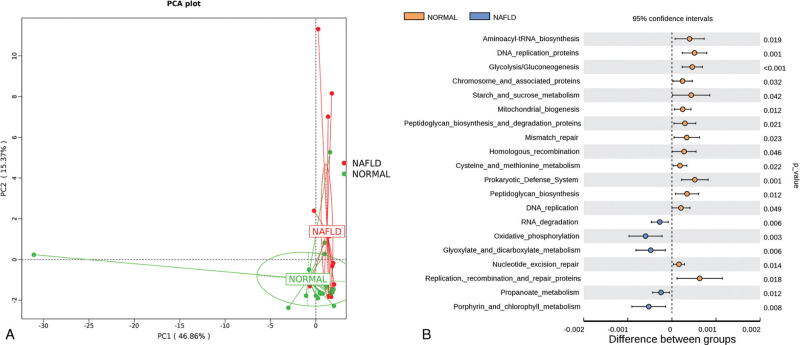
The prediction of gut microbiota phenotypes in NAFLD patients. (A) The PCA analysis of gut microbiota phenotypes of the Normal and NAFLD group. (B) The top 20 differential gut microbiota phenotypes identified by *t*-test analysis.

We used the Tax4Fun method to predict the phenotype of the gut microbiota. The phenotypic compositions of the normal and NAFLD groups were significantly different according to the PCoA (Fig. 2A). The top 20 differential phenotypes were analyzed by *t*-test and the biosynthesis of aminoacyl-tRNA was found to be the main phenotypic difference between the two groups. It was followed by peptidoglycan composition, metabolism of cysteine, methionine, glyoxylate, dicarboxylate, propanoate, porphyrin, and chlorophyll, glycolysis/gluconeogenesis, oxidative phosphorylation, and mitochondrial biogenesis (Fig. 2B). These results suggest that intestinal dysbacteriosis may be one of the causes of the amino acid, glycolipid, and energy metabolism related disorders in patients with NAFLD.

### 3.3 Gut microbiota network analysis

**Figure 3:**
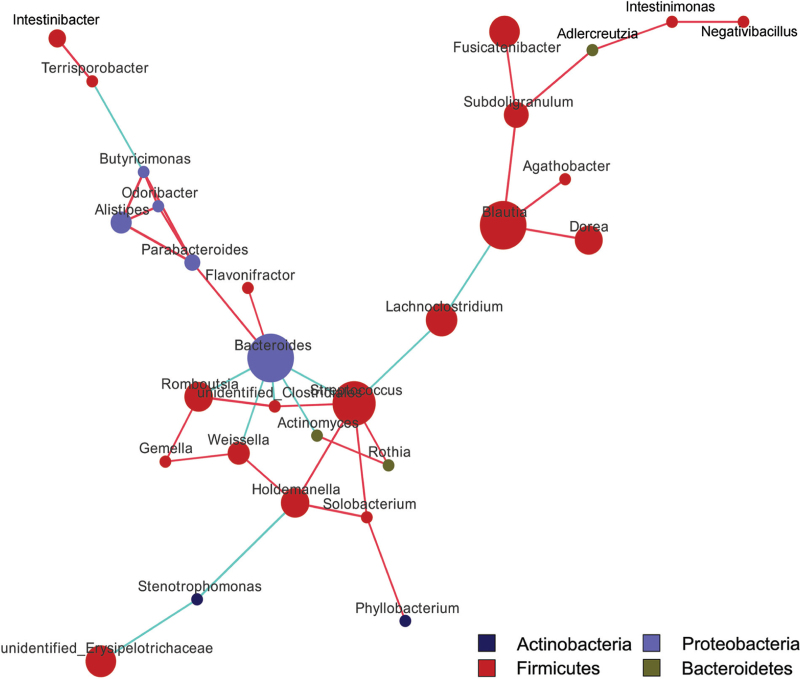
The gut microbiota network in NAFLD patients. The species co-occurrence of the gut microbiota in NAFLD patients is shown by the network. Spearman's correlation analysis was performed, and the genus was selected with *P* < .05, |r|> .6, and relative abundance > .005. Node represents genus, node size represents relative abundance, red line represents positive correlation, and blue line represents negative correlation.

We found that the dominant bacteria closely interact with other bacteria through species co-occurrence network in the intestinal tract of NAFLD patients (Fig. 5). Additionally, Firmicutes, Bacteroidetes, Proteobacteria, Bacteroides, Blautia, and Holdemanella are some of the dominant genera and found abundantly in the gut. They also play an important role in maintaining the microbial community structure and function owing to their presence in the center of species co-occurrence networks. Bacteroides and Holdmanelia were identified as the biomarkers of NAFLD by linear discriminant analysis effect size (LEfSe) analysis, further proving the role of these microbes in NAFLD progression (Fig. 3).

### 3.4 The metabolic characteristics in NAFLD

**Figure 4:**
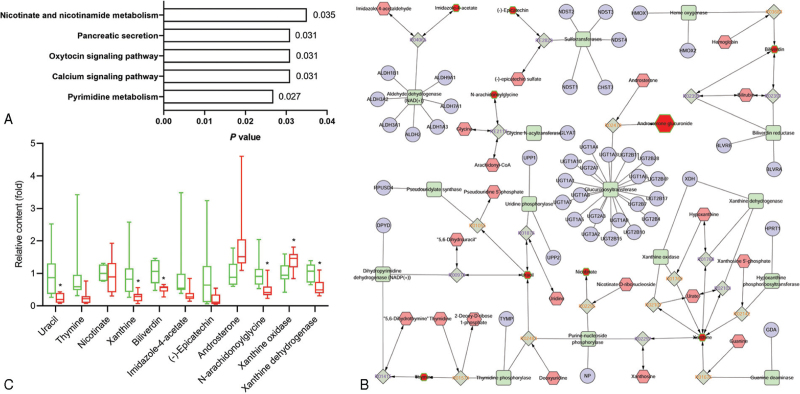
The analysis of metabolic characteristics in NAFLD patients. (A) The KEGG analysis of metabolic pathway in NAFLD patients feces. (B) The metabolite-enzyme network in NAFLD patients’ feces. (C) The different levels of metabolites and enzymes in healthy and NAFLD patients’ feces. ^∗^compared with Normal group, *P* < .05.

Metabolic analysis showed a significant change in fecal metabolite composition of NAFLD group. KEGG analysis showed that there were significant changes in the metabolism of Nicotinate, nicotinamide, and pyrimidine, signaling pathways of calcium and oxytocin, and pancreatic secretion (Fig. 4A), involving uracil, biliverdin, and xanthine, confirming the prediction of gut microbiota phenotypes. We also analyzed the above-mentioned metabolite-related enzymes, such as purine-nucleoside phosphorylase, glucuronosyltransferase, and aldehyde dehydrogenase, using the metabolite-enzyme network (Fig. 4B). Results demonstrated a significant decrease in the levels of uracil, xanthine, biliverdin, N-arachidonoylglycine, and xanthine dehydrogenase enzymes, and an increase in xanthine oxidase levels (Fig. 4C).

### 3.5 Biomarkers analysis of gut microbiota in NAFLD patients

**Figure 5:**
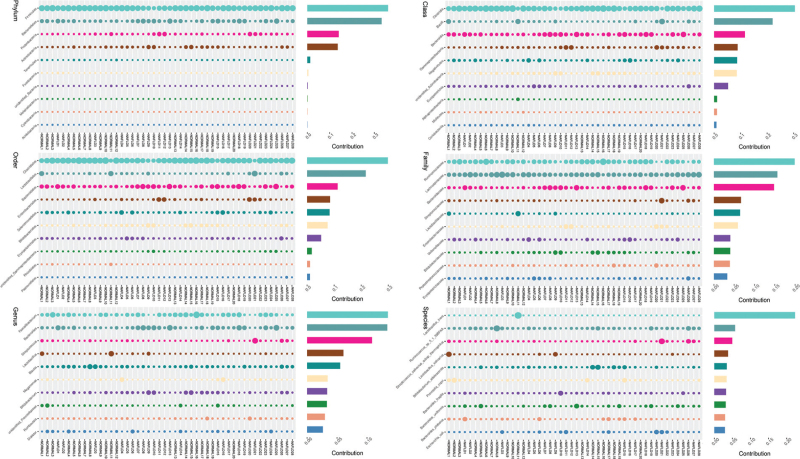
The contribution of gut microbiota in NAFLD patients. The contribution of the gut microbiota from the phylum to the species level was analyzed by similarity percentages in the normal and NAFLD groups.

**Figure 6:**
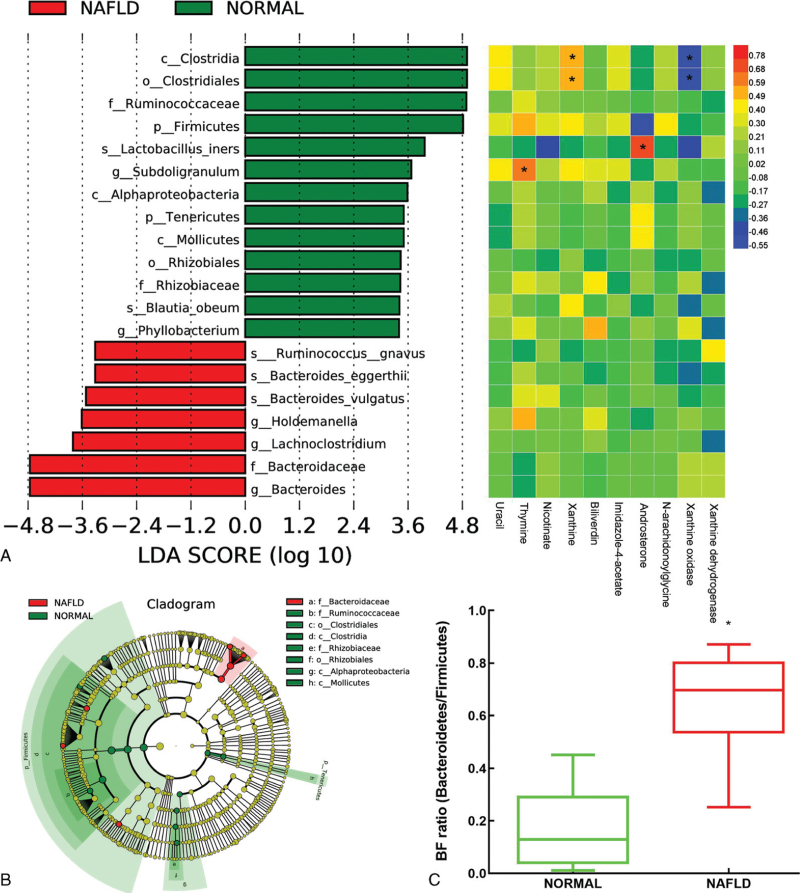
The biomarkers of gut microbiota in NAFLD patients. (A) The LEfSe analysis of gut microbiota of the Normal and NAFLD group and the correlation of gut microbiota biomarker and metabolite; (B) The evolutionary branch map of phylogenetic distribution of the gut microbiota biomarker species in Normal and NAFLD group; **(C)** The BF ratio (Bacteroides/Firmicutes) of Normal and NAFLD group.^∗^, compared with Normal group, *P* < .05.

The species abundance pattern of the normal and NAFLD groups was identified by analyzing the similarity percentages to determine the contribution of each species to the top 10 different species in the two groups, as shown in Figure 5. We used LEfSe analysis to identify the significantly different gut microbiota biomarkers in the normal and NAFLD groups. Moreover, their correlation with the metabolites such as xanthine, (-)-epicatechin, thymine, androsterone, and enzymes such as xanthine oxidase was established (Fig. 6A), which confirmed the prediction of the phenotype of the gut microbiota. The phylogenetic distribution of the biomarker species was shown by an evolutionary branch map suggesting that the biomarkers were mainly Firmicutes and Bacteroidetes in the normal and NAFLD groups, respectively (Fig. 6B). The BF ratio in the NAFLD group was higher than that of the normal group (Fig. 6C). We considered that the gut microbiota plays an important role in the metabolic characteristics of patients with NAFLD.

### 3.6 Diagnostic efficacy of gut microbiota in NAFLD

The area under the curve, sensitivity, specificity, and cutoff values were obtained by ROC analysis of the gut microbiota in the fecal samples (Table 2). Bacteroides and BF ratio had the highest sensitivity and specificity, respectively; therefore, they were analyzed in combination. Together, their sensitivity and specificity were 88% and 100%, respectively, which allows for a better index of diagnostic accuracy to be obtained.

**Table 2:** Diagnosis efficacy of gut microbiota in NAFLD.

Microbe	AUC	Sensitivity (%)	Specificity (%)	cut-off value
firmicutes	0.7018	43	90	0.6322
clostridia	0.7482	79	70	0.6231
clostridiales	0.7482	79	70	0.6231
ruminococcaceae	0.7554	68	80	0.2558
bacteroidaceae	0.7143	82	55	0.0564
bacteroides	0.7143	82	55	0.0564
lachnoclostridium	0.8241	61	95	0.0098
bacteroides_vulgatus	0.7330	57	85	0.0029
blautia_obeum	0.7125	50	95	0.0024
BF ratio	0.6625	36	100	0.4714

AUC = area under the curve.

## 4 Discussion

The incidence of NAFLD has increased significantly in recent years and has become a major cause of chronic liver disease worldwide, affecting human health adversely.^[9,10]^ In view of the gut-liver axis concept, gut microbiota has emerged as a crucial topic in liver disease research and demonstrated its impact on the occurrence and development of chronic liver disease. Despite advances in the gut microbiota macrogenomics, the characteristics of the NAFLD gut microbiota and its metabolites have not been fully defined.

The intestinal microecology is involved in the regulation of body metabolism and energy balance, including energy uptake, utilization, and consumption associated with carbohydrate and fat metabolism.^[11]^ Research findings have shown that the human gut microbiota can produce a variety of metabolites including short chain fatty acids, such as acetic acid, propionic acid, and butyric acid, which are produced by the decomposition of undigested dietary components.^[12]^ Moreover, vitamin B and K affect the metabolism of the human body, thereby playing significant roles in pathogenesis of obesity, metabolic syndrome, diabetes, NAFLD, allergic reaction, inflammatory bowel disease and tumorigenesis in case of intestinal microecological imbalance.^[13]^

The gut microbiota, internal and external environment of the healthy human body always maintain a dynamic balance.^[14]^ An imbalance in the intestinal microecological structure may lead to the development of obesity and related metabolic diseases, which is evident in the present study of patients with NAFLD.^[15]^ Compared to healthy subjects, the microbial community structure in fecal samples of NAFLD patients is quite different with less diversity of microbial communities in the gut. Studies have shown that normal gut microbiota diversity is crucial for a healthy metabolism and changes in this factor can induce many diseases, such as hepatic cirrhosis, diabetes mellitus, and tumors. Therefore, an imbalance in the gut microbiota may be a potential cause of NAFLD.

The present study found that Bacteroides, Blautia, and Holdmanelia were the most abundant genera in the feces of patients with NAFLD. The LEfSe analysis revealed a significant difference in their relative abundance from that of the healthy population; therefore, they may be useful as potential NAFLD biomarkers. According to the previous reports, increased abundance of Bacteroides is associated with NAFLD and positively correlated with the severity of NAFLD, which is consistent with the findings of this study.^[16,17]^ Additionally, LEfSe analysis revealed that Firmicutes can serve as important biomarkers in the NAFLD group, due to their significant down regulation in contrast to Bacteroides. Therefore, the BF ratio can be used as an index of obesity. The BF ratio in obese people is significantly higher than that in people with normal body weight, indicating the potential role of dysregulated gut microbiota in causing obesity.^[18]^ We observed significantly higher BF ratios in NAFLD patients than those in healthy subjects; therefore it could be used as a potential basis for NAFLD diagnosis.

Firmicutes is an important part of the gut microbiota due to its role in promoting the effective absorption of nutrients from food and their conversion into fat. Due to its strong correlation with human sugar metabolism, decreased Firmicutes could lead to an abnormal glycolipid metabolism.^[19]^ Blautia is one of the most abundant bacteria in the gut, producing butyric acid and acetic acid.^[20]^ Kimura et al suggested that it can reduce obesity by regulating G protein-coupled receptors 41 and 43, and has potential to maintain or improve the metabolic syndrome-related disease status.^[21]^ Holdmanelia are aerobic or facultative anaerobic, non-fermented, Gram-negative bacilli. They can oxidize and decompose mono-, di-, and polysaccharides, and use them as only carbon and nitrogen sources to produce short chain fatty acids.^[22]^

The above findings reveal that Firmicutes, Bacteroides, Blautia, and Holdmanelia are not only potential biomarkers for NAFLD, but can also influence its progression by regulating glycolipid metabolism, which is consistent with the results of flora phenotype prediction. Previous studies and phenotypic prediction in present study suggest that gut microbiota can influence the NAFLD process by regulating metabolism of various molecules, such as starch, sucrose, cysteine, methionine, glyoxylate, and dicarboxylate. KEGG analysis showed that nicotinate, nicotinamide, and pyrimidine metabolism, calcium and oxytocin signaling pathways, and pancreatic secretion were altered in the fecal samples of NAFLD patients. The above-mentioned pathways are important metabolic pathways involving sugar, lipid, and oxidative reduction, which cause NAFLD in case of any dysregulation.

We also found a correlation between various genera including Clostridia, Clostridiales, and Blautia obeum and the metabolites, such as nicotinate, androsterone glucuronide, and xanthine, indicating a potential relationship between gut microbiota and abnormal metabolism in NAFLD by metabolomics and ELISA assays.

Xanthine dehydrogenase and xanthine oxidase are important enzymes involved in regulating blood free fatty acid levels and oxidative stress.^[23,24]^ Since liver is the main organ involved in glycolipid metabolism, any dysregulation of this process may lead to NAFLD. Xanthine dehydrogenase decreases oxidative stress and increases insulin sensitivity whereas, xanthine oxidase plays an opposite role.^[25,26]^ Thus, the balance of these two enzymes plays an important role in inhibiting NAFLD. In present study, levels of xanthine dehydrogenase were significantly decreased while those of xanthine oxidase were increased in the feces of NAFLD patients. Also, xanthine oxidase levels were correlated with Clostridia and Clostridiales, suggesting that it may be a key target of gut microbiota to regulate glycolipid metabolism.

At present, liver biopsy remains the standard diagnostic tool for NAFLD. Due to its invasive procedure, high-cost, and other limitations, alternate methods are required in clinical practice. Abdominal ultrasound and biochemistry are the most commonly used methods to check for liver steatosis; however, there is still some subjectivity. To devise a simple NAFLD diagnostic method, this study established that Bacteroides and Firmicutes offer a certain diagnostic accuracy as determined by ROC curve analysis. The BF ratio combined with Bacteroides had a sensitivity of 88% and a specificity of 100%. This index can provide an auxiliary value for NAFLD diagnosis.

## 5 Conclusion

The structure and phenotype of the gut microbiota in patients with NAFLD change significantly. In comparison with previous studies, we found that xanthine and related enzymes serve as an important link between gut microbiota and NAFLD. Additionally, it may be a significant tool in exploring the combination of Bacteroides and BF ratio for NAFLD diagnostic accuracy and its mechanism. The mechanism of gut microbiota dysbacteriosis-induced NAFLD can be further investigated by animal experiments. Due to small sample size of our study, the feasibility of using gut microbiota as a NAFLD diagnostic approach should be studied in a larger sample size. These findings may provide new therapeutic targets and diagnostic techniques for NAFLD treatment.

## Author contributions

SL and WW were responsible for study conception and design. HG, HC, and YT were responsible for data acquisition. YZ, YL, XS, and SL were responsible for data interpretation. All authors have read and approved the final manuscript.
